# Supplementary material for: Characteristics of inflammatory response and repair after experimental blast lung injury in rats
Source: PLoS One. 2023 Mar 16;18(3):e0281446. doi: 10.1371/journal.pone.0281446 (PMC10019677; doi:10.1371/journal.pone.0281446)
Supplement: S1 Fig — (PPT) [file pone.0281446.s001.ppt]

## Slide 1
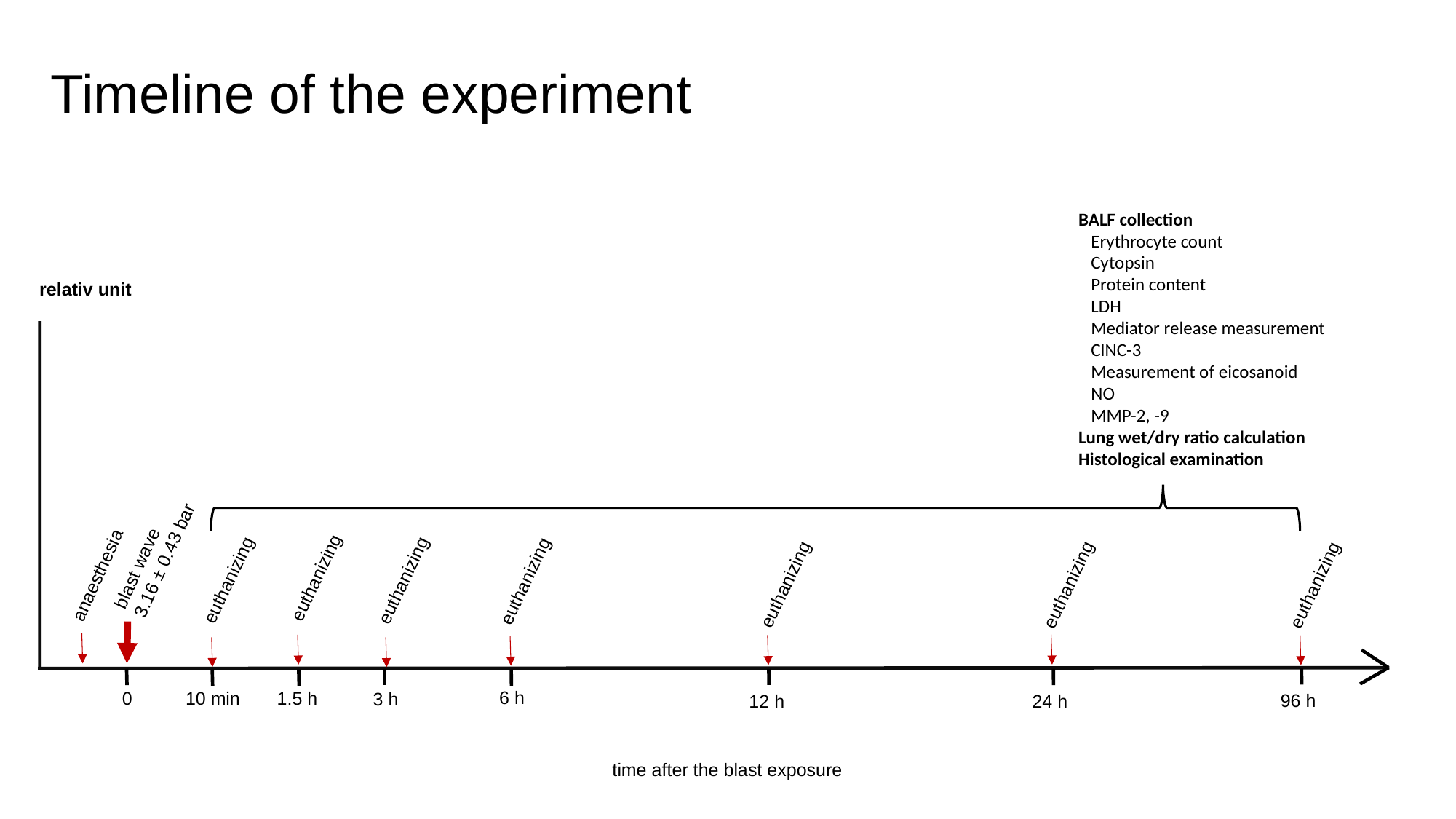

Timeline of the experiment
BALF collection
 Erythrocyte count
 Cytopsin
 Protein content
 LDH
 Mediator release measurement
 CINC-3
 Measurement of eicosanoid
 NO
 MMP-2, -9
Lung wet/dry ratio calculation
Histological examination
relativ unit
blast wave
3.16 ± 0.43 bar
anaesthesia
 euthanizing
 euthanizing
 euthanizing
 euthanizing
 euthanizing
 euthanizing
 euthanizing
6 h
10 min
1.5 h
0
3 h
96 h
12 h
24 h
time after the blast exposure
