# Supplementary material for: Characteristics of inflammatory response and repair after experimental blast lung injury in rats
Source: PLoS One. 2023 Mar 16;18(3):e0281446. doi: 10.1371/journal.pone.0281446 (PMC10019677; doi:10.1371/journal.pone.0281446)
Supplement: S1 File — (DOC) [file pone.0281446.s002.doc]

**The ARRIVE Essential 10**

These items are the basic minimum to include in a manuscript. Without this information, readers and reviewers cannot assess the reliability of the findings.

**Study design**

1 For each experiment, provide brief details of study design including:

a. The groups being compared, including control groups. If no control group has been used, the rationale should be stated.

Rats underwent a standardized blast wave trauma and were euthanised at defined time points. Non-traumatised animals served as sham controls. Samples like lung tissue, bronchoalveolar lavage fluid or blood were assessed at each time-point were assessed for outcomes like inflammatory parameters or cytokine/chemokine profile.

b. The experimental unit (e.g. a single animal, litter, or cage of animals).

**Sample size**

2 a. Specify the exact number of experimental units allocated to each group, and the total number in each experiment. Also indicate the total number of animals used.

Usually in the setting of explorative analyses without background of former experiments and no former eximerimental ananlysis allowing sample size calculation, a number of 6 end results per group were the aim. We could therefore not base in the experimental settings of this work on a power analysis for testing the number of animals.

b. Explain how the sample size was decided. Provide details of any a priori sample size calculation, if done.

Same as 2 a.

**Inclusion and exclusion criteria**

3 a. Describe any criteria used for including and excluding animals (or experimental units) during the experiment, and data points during the analysis. Specify if these criteria were established a priori. If no criteria were set, state this explicitly.

No further criteria were used.

b. For each experimental group, report any animals, experimental units or data points not included in the analysis and explain why. If there were no exclusions, state so.

Only rats who died before the end point were excluded. No further exclusions were performed.

c. For each analysis, report the exact value of n in each experimental group.

Except experiment on thorax trauma-induced weight gain, which resulted the figure 6, in the rest of experiments animal numbers have been specified. In this data the n is therefore ≥ 6.

**Randomisation**

4 a. State whether randomisation was used to allocate experimental units to control and treatment groups. If done, provide the method used to generate the randomisation sequence.

No randomisation was performed, but the animal experiments were performed in iterative change with end point.

b. Describe the strategy used to minimise potential confounders such as the order of treatments and measurements, or animal/cage location. If confounders were not controlled, state this explicitly.

The rats were allocated iteratively to end points, but not in terms of a randomizaton list, to obtain minimal bias in the animal study groups.

**Blinding**

5 Describe who was aware of the group allocation at the different stages of the experiment (during the allocation, the conduct of the experiment, the outcome assessment, and the data analysis).

No blinding were performed.

**Outcome measures**

6 a. Clearly define all outcome measures assessed (e.g. cell death, molecular markers, or behavioural changes).

All outcome measures have been presented in the submitted text.

b. For hypothesis-testing studies, specify the primary outcome measure, i.e. the outcome measure that was used to determine the sample size.

The work was not a hypothesis-testing study.

**Statistical methods**

7 a. Provide details of the statistical methods used for each analysis, including software used.

Statistical methods for every analysis have been separatedly mentioned and statistics were performed with the Graph Pad Prism 3.0 software (Graph Pad Software, San Diego, CA, USA). Every statistical analysis was performed using the control features for distibution testing of the software, therefore, all tests based on normal distribution were always verified by this step. Notably, we have done all the controls needed to use the parametric tests.

b. Describe any methods used to assess whether the data met the assumptions of the statistical approach, and what was done if the assumptions were not met.

See 7 a.

**Experimental animals**

8 a. Provide species-appropriate details of the animals used, including species, strain and substrain, sex, age or developmental stage, and, if relevant, weight.

Female Wistar rats (Weight 230 ± 20 g; Harlan Winkelman GmbH, Borchen, Germany) were used in all experiments.

b. Provide further relevant information on the provenance of animals, health/immune status, genetic modification status, genotype, and any previous procedures.

The animals were housed in a central specific-pathogen-free animal house facility. Animals were fed standard rat chow and had access to water ad libitum until the start of the experiment.

**Experimental procedures**

9 For each experimental group, including controls, describe the procedures in enough detail to allow others to replicate them, including:

a. What was done, how it was done and what was used.

b. When and how often.

c. Where (including detail of any acclimatisation periods).

d. Why (provide rationale for procedures).

All above mentioned criteria have been described in our experiments.

**Results**

10 For each experiment conducted, including independent replications, report:

a. Summary/descriptive statistics for each experimental group, with a measure of variability where applicable (e.g. mean and SD, or median and range).

b. If applicable, the effect size with a confidence interval.

The descriptive statistics as indicated have been given in all results.

**The Recommended Set**

These items complement the Essential 10 and add important context to the study. Reporting the items in both sets represents best practice.

**Abstract**

11 Provide an accurate summary of the research objectives, animal species, strain and sex, key methods, principal findings, and study conclusions.

**Objective**: The objective of the current study was to define sequence of such alterations in with establishing blast-induced lung injury in rats using an advanced blast generator.

**Methods**: Rats underwent a standardized blast wave trauma and were euthanised at defined time points. Non-traumatised animals served as sham controls. Obtained samples from bronchoalveolar lavage fluid (BALF) at each time-point were assessed for histology, leukocyte infiltration and cytokine/chemokine profile.

**Results:** After blast lung injury, significant haemorrhage and neutrophil infiltration were observed. Similarly, protein accumulation, lactate dehydrogenase activity (LDH), alveolar eicosanoid release, matrix metalloproteinase(MMP)-2 and -9, pro-Inflammatory cytokines, including tumour necrosis factor (TNF) and interleukin (IL) -6 raised up. While declining in the level of anti-inflammatory cytokine IL-10 occurred. Ultimately, pulmonary oedema developed that increased to its maximum level within the first 1.5 h, then recovered within 24 h.

**Conclusion:** Using a stablished model, can facilitate the study of inflammatory response to blast lung injury. Following the blast injury, alteration in cytokine/chemokine profile and activity of cells in the alveolar space occurs, which eventuates in alveolar epithelial barrier dysfunction and oedema formation. Most of these parameters exhibit time-dependent return to their basal status that is an indication to resilience of lungs to blast-induced lung injury.

**Background**

12 a. Include sufficient scientific background to understand the rationale and context for the study, and explain the experimental approach.

Scientific background has been explained in introduction and it fairly justified the rationale and context for conducting the study. Experimental approach have been described in detail in matherial and methods.

b. Explain how the animal species and model used address the scientific objectives and, where appropriate, the relevance to human biology.

**Objectives**

13 Clearly describe the research question, research objectives and, where appropriate, specific hypotheses being tested.

Research question: What is the characteristics of inflammatory response and repair after experimental blast lung injury in rats

Research question: To define the characteristics of inflammatory response and repair after experimental blast lung injury in rats

Specific hypotheses being tested:

The basis of the work is descriptive; hypothesis testing has not been a key component.

**Ethical statement**

14 Provide the name of the ethical review committee or equivalent that has approved the use of animals in this study, and any relevant licence or protocol numbers (if applicable). If ethical approval was not sought or granted, provide a justification.

The study was approved by the Federal Animal Care and Use Committee Tübingen, Germany.

**Housing and husbandry**

15 Provide details of housing and husbandry conditions, including any environmental enrichment.

The animals were housed in a central specific-pathogen-free animal house facility under the oversight of the institutional animal welfare officer. Animals were fed standard rat chow and had access to water ad libitum until the start of the experiment.

**Animal care and monitoring**

16 a. Describe any interventions or steps taken in the experimental protocols to reduce pain, suffering and distress.

Before blast expousure, the rats were anaesthetised in a bell jar flooded with a Halothane® (Sigma-Aldrich Chemie GmbH, Deisenhofen, Germany)/O2 gas mixture through a Halothane-evaporator (Flow: 2 L/min oxygen, 4% Halothane).

After blast also, ratss were terminally anaesthetised by an intraperitoneal injection of sodium pentobarbital (Narcoren®, 160 mg/kg BW).

b. Report any expected or unexpected adverse events.

c. Describe the humane endpoints established for the study, the signs that were monitored and the frequency of monitoring. If the study did not have humane endpoints, state this.

**Interpretation/scientific implications**

17 a. Interpret the results, taking into account the study objectives and hypotheses, current theory and other relevant studies in the literature.

It has been done in discussion section.

b. Comment on the study limitations including potential sources of bias, limitations of the animal model, and imprecision associated with the results.

Our model is associated with a number of limitations. Data from human patients rather than animal experiments are required. In addition, thoracic trauma deteriorates lung function depending on the trauma intensity. On the other hand, the airway mechanics of traumatized rat lungs appear to be more susceptible to ex vivo mechanical ventilation and perfusion. Hence, the probable development of a secondary injury (ventilator-associated lung injury) should not be overlooked as another limitation of the study.

**Generalisability/translation**

18 Comment on whether, and how, the findings of this study are likely to generalise to other species or experimental conditions, including any relevance to human biology (where appropriate).

The present study established a blast-induced lung injury model in rats, which provides valid and reproducible results. It might facilitate future research on the modulation of blast lung injury in view of the major impact of trauma on especially young patients and thus on society. In addition to the time-dependent resolution of the trauma-related alveolar protein-rich fluid, these results gave evidence of the high repair including the alveolar fluid, cell, and cell debris clearance capacity of the lung.

**Protocol registration**

19 Provide a statement indicating whether a protocol (including the research question, key design features, and analysis plan) was prepared before the study, and if and where this protocol was registered.

The work has been a biological doctorate thesis that was aimed to give insight into the pathogenesis of acute lung injury and inflammatory and its pro-and antioedematous aspects. There was no protocol registration for this study.

**Data access**

20 Provide a statement describing if and where study data are available.

The presented work is part of a thesis that is available in the internet.

**Declaration of interests**

21 a. Declare any potential conflicts of interest, including financial and non-financial. If none exist, this should be stated.

Competing Interests statement

The work of YH, UM, and JH have been funded by Lungen- und Atmungsstiftung Bern, Switzerland which provides non-restricted financial support toward research on lung, respiratory and sleep-related respiratory diseases as well as related rehabilitation and lifestyle change issues such as exercise, smoking cessation, etc. JH is the chair of Lungen- und Atmungsstiftung Bern and his position did not influence the design of the study, the collection of the data, the analysis or interpretation of the data, the decision to submit the manuscript for publication, or the writing of the manuscript and did not present any financial conflicts. Also, the work of JH was supported by a grant from the Deutsche Forschungsgemeinschaft (FOR 321/2-1; research group “Endogenous tissue injury: Mechanisms of autodestruction”) and by the Herrmann Josef Schieffer Prize of the “Freunde des Universitätsklinikums Homburg e.V.”. All remaining authors (HH, LB, US, AW and RL) declare no potential financial or non-financial conflict of interest with the work presented here. This does not alter our adherence to PLOS ONE policies on sharing data and materials.

b. List all funding sources (including grant identifier) and the role of the funder(s) in the design, analysis and reporting of the study.

Fundation provided by Lungen- und Atmungsstiftung Bern, Switzerland and grant from the Deutsche Forschungsgemeinschaft (FOR 321/2-1; research group “Endogenous tissue injury: Mechanisms of autodestruction”) and by the Herrmann Josef Schieffer Prize of the “Freunde des Universitätsklinikums Homburg e.V.

Funders perform no role in the design, analysis and reporting of the study.
