# Supplementary material for: Characteristics of inflammatory response and repair after experimental blast lung injury in rats
Source: PLoS One. 2023 Mar 16;18(3):e0281446. doi: 10.1371/journal.pone.0281446 (PMC10019677; doi:10.1371/journal.pone.0281446)
Supplement: Attachment — Submitted filename: Response to Reviewers_inflammatory response_004_220822_YH.doc [file pone.0281446.s003.doc]

# Responses to Reviewer’s comments: Characteristics of inflammatory response and repair after experimental blast lung injury in rats

PONE-D-22-09944

1. A marked-up copy of your manuscript that highlights changes made to the original version. You should upload this as a separate file labeled 'Revised Manuscript with Track Changes'.
2. An unmarked version of your revised paper without tracked changes. You should upload this as a separate file labeled 'Manuscript'.

When submitting your revision, we need you to address these additional requirements.

3. Please ensure that your manuscript meets PLOS ONE's style requirements, including those for file naming. The PLOS ONE style templates can be found at <https://journals.plos.org/plosone/s/file?id=wjVg/PLOSOne_formatting_sample_main_body.pdf> and <https://journals.plos.org/plosone/s/file?id=ba62/PLOSOne_formatting_sample_title_authors_affiliations.pdf>
4. We suggest you thoroughly copyedit your manuscript for language usage, spelling, and grammar. If you do not know anyone who can help you do this, you may wish to consider employing a professional scientific editing service. Upon resubmission, please provide the following: The name of the colleague or the details of the professional service that edited your manuscript

We acknowledge that Dr Rudolf Lucas who works since > 15 y in the USA co-wrote and several times revised the text and that we therefore think that language is acceptable.

1. A copy of your manuscript showing your changes by either highlighting them or using track changes (uploaded as a *supporting information* file)
2. A clean copy of the edited manuscript (uploaded as the new *manuscript* file)
3. As part of your revision, please complete and submit a copy of the Full ARRIVE 2.0 Guidelines checklist, a document that aims to improve experimental reporting and reproducibility of animal studies for purposes of post-publication data analysis and reproducibility:  [https://arriveguidelines.org/sites/arrive/files/Author%20Checklist%20-%20Full.pdf](https://arriveguidelines.org/sites/arrive/files/Author Checklist - Full.pdf) (PDF). Please include your completed checklist as a Supporting Information file. Note that if your paper is accepted for publication, this checklist will be published as part of your article.
4. Thank you for submitting the above manuscript to PLOS ONE. During our internal evaluation of the manuscript, we found significant text overlap between your submission and the following previously published works, some of which you are an author.<https://d-nb.info/969514263/34>. We would like to make you aware that copying extracts from previous publications, especially outside the methods section, word-for-word is unacceptable. In addition, the reproduction of text from published reports has implications for the copyright that may apply to the publications. Please revise the manuscript to rephrase the duplicated text, cite your sources, and provide details as to how the current manuscript advances on previous work. Please note that further consideration is dependent on the submission of a manuscript that addresses these concerns about the overlap in text with published work. We will carefully review your manuscript upon resubmission, so please ensure that your revision is thorough.
5. We note that the grant information you provided in the ‘Funding Information’ and ‘Financial Disclosure’ sections do not match. When you resubmit, please ensure that you provide the correct grant numbers for the awards you received for your study in the ‘Funding Information’ section.

The work of YH, UM, and JH have been funded by Lungen- und Atmungsstiftung Bern, Switzerland which provides non-restricted financial support toward research on lung, respiratory and sleep-related respiratory diseases as well as related rehabilitation and lifestyle change issues such as exercise, smoking cessation, etc. JH is the chair of Lungen- und Atmungsstiftung Bern and his position did not influence the design of the study, the collection of the data, the analysis or interpretation of the data, the decision to submit the manuscript for publication, or the writing of the manuscript and did not present any financial conflicts. Also, the work of JH was supported by a grant from the Deutsche Forschungsgemeinschaft (FOR 321/2-1; research group “Endogenous tissue injury: Mechanisms of autodestruction”) and by the Herrmann Josef Schieffer Prize of the “Freunde des Universitätsklinikums Homburg e.V.”. All remaining authors (HH, LB, US, AW and RL) declare no potential financial or non-financial conflict of interest with the work presented here.

6. Thank you for stating the following in the Competing Interests section:
   "The work of YH, UM, and JH have been funded by Lungen- und Atmungsstiftung Bern, Switzerland which provides non-restricted financial support toward research on lung, respiratory and sleep-related respiratory diseases as well as related rehabilitation and lifestyle change issues such as exercise, smoking cessation, etc. JH is the chair of Lungen- und Atmungsstiftung Bern and his position did not influence the design of the study, the collection of the data, the analysis or interpreta-tion of the data, the decision to submit the manuscript for publication, or the writing of the manuscript and did not present any financial conflicts. Also, the work of JH was supported by a grant from the Deutsche Forschungsgemeinschaft (FOR 321/2-1; research group “Endogenous tissue injury: Mechanisms of autodestruction”) and by the Herrmann Josef Schieffer Prize of the “Freunde des Universitätsklinikums Homburg e.V.”. All remaining authors (HH, MM, LB, US, AW and RL) declare no potential financial or non-financial conflict of interest with the work presented here."Please confirm that this does not alter your adherence to all PLOS ONE policies on sharing data and materials, by including the following statement: ""This does not alter our adherence to  PLOS ONE policies on sharing data and materials.” (as detailed online in our guide for authors <http://journals.plos.org/plosone/s/competing-interests>).  If there are restrictions on sharing of data and/or materials, please state these. Please note that we cannot proceed with consideration of your article until this information has been declared.

We confirm here that this does not alter our adherence to all PLOS ONE policies on sharing data and materials, by including the following statement. It has been also mentioned in the manuscript.

7. Please include your updated Competing Interests statement in your cover letter; we will change the online submission form on your behalf.

Thank you for this comment. It has been done.

8. PLOS ONE now requires that authors provide the original uncropped and unadjusted images underlying all blot or gel results reported in a submission’s figures or Supporting Information files. This policy and the journal’s other requirements for blot/gel reporting and figure preparation are described in detail at [https://journals.plos.org/plosone/s/figures#loc-blot-and-gel-reporting-requirements](https://journals.plos.org/plosone/s/figures#loc-blot-and-gel-reporting-requirements) and [https://journals.plos.org/plosone/s/figures#loc-preparing-figures-from-image-files](https://journals.plos.org/plosone/s/figures#loc-preparing-figures-from-image-files). When you submit your revised manuscript, please ensure that your figures adhere fully to these guidelines and provide the original underlying images for all blot or gel data reported in your submission. See the following link for instructions on providing the original image data: [https://journals.plos.org/plosone/s/figures#loc-original-images-for-blots-and-gels](https://journals.plos.org/plosone/s/figures#loc-original-images-for-blots-and-gels).

Thank you for this comment. We have removed figure 14 as we do not have the certainty that it was an elaborated gel and we do not have access to control the original gel.

9. In your cover letter, please note whether your blot/gel image data are in Supporting Information or posted at a public data repository, provide the repository URL if relevant, and provide specific details as to which raw blot/gel images, if any, are not available. Email us at [plosone@plos.org](mailto:plosone@plos.org) if you have any questions.
10. We note that you have included the phrase “data not shown” in your 11. manuscript. Unfortunately, this does not meet our data sharing requirements. PLOS does not permit references to inaccessible data. We require that authors provide all relevant data within the paper, Supporting Information files, or in an acceptable, public repository. Please add a citation to support this phrase or upload the data that corresponds with these findings to a stable repository (such as Figshare or Dryad) and provide and URLs, DOIs, or accession numbers that may be used to access these data. Or, if the data are not a core part of the research being presented in your study, we ask that you remove the phrase that refers to these data.

We have removed the phrase “data not shown” and deleted/modified its related sections.

[NOTE: If reviewer comments were submitted as an attachment file, they will be attached to this email and accessible via the submission site. Please log into your account, locate the manuscript record, and check for the action link "View Attachments". If this link does not appear, there are no attachment files.]

While revising your submission, please upload your figure files to the Preflight Analysis and Conversion Engine (PACE) digital diagnostic tool, <https://pacev2.apexcovantage.com/>. PACE helps ensure that figures meet PLOS requirements. To use PACE, you must first register as a user. Registration is free. Then, login and navigate to the UPLOAD tab, where you will find detailed instructions on how to use the tool. If you encounter any issues or have any questions when using PACE, please email PLOS at [figures@plos.org](mailto:figures@plos.org). Please note that Supporting Information files do not need this step.

### 5. Review Comments to the Author

Please use the space provided to explain your answers to the questions above. You may also include additional comments for the author, including concerns about dual publication, research ethics, or publication ethics. (Please upload your review as an attachment if it exceeds 20,000 characters)

**Reviewer #1:** The article by Hamacher et al. concerns the characterization of inflammatory response an repair after experimental blast injury.They showed that following blast injury an alteration of cytokines and activity cells is observed. They showed a neutrophil infiltration into the alveolar space, signature of inflammation. The study is well conduct but I would like to mention that I am not specialized in blast injury.

I have some comments.

1. Figure 2 is not mentioned in the text as figure 3 (results paragraph e3.2, Figure 3C is mentioned instead of figure 2).

The reviewer is right, now we have corrected figures’ numbers.

2. What is the number of animals per group? 1? n is the number of experiments ? one experiment is one animal /group ?

In every paragraph the numbers of animals per experiment has been indicated.

3. Regarding the statistics, I am quite surprised because the results are expressed as an mean +/- SEM. SEM is important. It would be interesting to represent the results as a scatter plot to show the individual response of all animals.

Unfortunately we have not the original data any more in our hands and are therefore not able to re-plot the graphs in scatter plots. We asked the university of Konstanz to have access to the original data, but have not yet got this access as all those laboratory books including all data from assays are given in those books. Unfortunately so far we have not had this access.

4. For figure 10, the points are connected, which is not acceptable because the animals are not the same for each time. This figure must be modified and represented like the others.

Reviewer is totally right. We have modified this figure.

5. I suggest to include the MPO result with neutrophils paragraph.

Thank you for this comment. It has been done.
For the MMP part (results) it could be shorter by extracting a part already integrated in the discussion.

Thank you for this comment. Now, it has been done.

6. The discussion is rather long and not concise enough. It's confusing. Why not discuss the elements as a whole. For example, the discussion on TNF is in three paragraphs or even 4 and quite long and a bit redundant. PMN, MMP, CINC could be discussed together in sub-paragraphs.

**Reviewer #2:** This manuscript dealing with post blast lung inflammatory response presents results of interest for the scientific community. However, numerous issues prevent this manuscript being published as is.
Global remarks:

1- the manuscript has to be professionally language edited

We acknowledge that Dr. Rudolf Lucas who works since > 15 y in the USA co-wrote and several times revised the text and that we therefore think that language is acceptable.

2- the figure's numbers are incorrect

The reviewer is totally right. Now the figure’s numbersa have been corrected.

3- citations are sometimes not cited correctly

Thank you for this comment. They have been corrected and corrected.

Specific remarks:

4- the statistics are using parametric tests with very small n in each groups. Non parametric tests need to be used. Also, when using small groups, data must be presented as Median [IQR] instead of mean (SEM or SD)

We always were attentive to check whether we had to do non-parametric testing. In all the dataset we are confident that we did not violate the rules for parametric tests. We otherwise would have given the data and results with median and ranges and would have indicated the non-parametric test methods.

5- the authors does not discuss the similarities/differences with a classical blunt trauma model. An interesting question should be: How the blast related lung injuries are different from blunt chest trauma related lung injuries?

The reviewer is right that we did not discuss. We discussed it in the discussion section as: Blast thoracic injury seems to be the most dangerous type of non-penetrating thoracic injury that may more regularly lead to death due to acute lung injury than in other non-penetrating thoracic injuries (CDC Explosions and blast injuries. A primer for Clinicians).

6- thus, the definition of the incoming blast overpressure wave is of importance. The OP wave provided by the shock tube is very short (0.6 ms) where standout open field OP waves are close to 2-4 ms. It is understandable that even if very short, this OP wave is responsible for lung injuries, but this singularity is not discussed in the paper. how short OP wave related lung injury is different from normal length OP wave related lung injury?

The question is for sure of physical and of pathophysiological importance. We had a clear and defined experimental setting, also certainly with minimal distance to the subject, and we have no clear clue to alternative and slower overpressure waves. Unfortunately, we can therefore not answer the question.

7- the wet to dry ratio study is of interest but it does not take the intra-alveolar hemorrhage into account. Also, methods don't describe if W/D ratio lungs were previously injected for BAL... The methods need to be clearer.

The wet to dry ratio is a standardized procedure performed in acute lung injury research since decades. Every clinically relevant acute lung injury is accompanied with some degree of destruction of the alveolar-capillary barrier and therefore of alveolar haemorrhage. This is e.g. very well seen in the scientific work on clinical acute lung injury and ARDS work where bronchoalveolar lavages have been analysed. The only point is that there it is in many times not quantified, but it is present. Therefore in virtually all data on severe acute lung injury some alveolar haemorrhage is present. We therefore are confident that this does not inferiorize the dataset presented on an injury where about 10 % of rats died within a few minutes thereafter. We consider it as a normal phenomenon not disturbing the value of wet to dry ratio.

8- Concerning the methods, why is the anesthetic strategy biphasic (firs halothan then pentothal) ? Also the pro-inflammatory effect of buprenorphine is not discussed.

The anaesthetic strategy was also given by the ethics committee, as in animal research the main focus is - e. g. besides reduction of animal numbers – to give the animals intravitally minimal harm. We therefore had to cover the whole intravital research for the rats with maximal analgesia, and therefore this anaesthetic strategy had to be chosen. Neither pain or dyspnea itself, nor an analgetic drug is probably completely free of biologic effects, and therefore any way with more or less pain and with any major analgetic drug may modify to a certain extent the read-outs as e.g. discussed by you.

This dilemma of animal in vivo research is in many instances best solved with rather generous analgetics and anaesthetics, and with the use of sham controls, as it is done only with very few scientifically and ethically acceptably exceptions in the animal research and its moral obligation of humane care.

The literature on buprenorphine is impressive and shows a number of anti-as a number of prl-inflammatory research papers.

We integrated in the limitation section one sentence on the possible effect of buprenorphine concerning the influence on outcome.
