# Supplementary material for: Characteristics of inflammatory response and repair after experimental blast lung injury in rats
Source: PLoS One. 2023 Mar 16;18(3):e0281446. doi: 10.1371/journal.pone.0281446 (PMC10019677; doi:10.1371/journal.pone.0281446)
Supplement: Attachment — Submitted filename: Response to Reviewers_003_230113_YH.doc [file pone.0281446.s004.doc]

**Response to Reviewers**

6. Review Comments to the Author

Please use the space provided to explain your answers to the questions above. You may also include additional comments for the author, including concerns about dual publication, research ethics, or publication ethics. (Please upload your review as an attachment if it exceeds 20,000 characters)

Reviewer #2: We thank the authors for their responses, however some points needs to be clarified in the manuscript.
**Point 1-3**: ok
**Point 4** – Statistics.
The authors state in their response and in the ARRIVE document that they did all the controls needed to use the parametric tests. This have to be also stated in the manuscript, section 2.6.

Thank you very much for this comment. In the section 2.6., and ARRIVE document we mentioned that "Notably, we have done all the controls needed to use the parametric tests."

**Point 5:** Comparison with blunt thoracic trauma. The authors add a comparison with blunt trauma concerning the general severity. The manuscript would benefit from an addition on the difference, even if poorly known, on the pathophysiological aspect of the lung contusion formation between the two different sorts of insult.

The shock wave produced by explosions and high-velocity projectiles may cause serious trauma to the pulmonary parenchyma. The onset of damage from a blast wave occurs when the blast wave hits the thoracic wall and compresses thorax and intrathoracic tissues and thus, in particular, the intrathoracic gas volume. The resulting forces exceed the tensile strength of the tissues and damage their structural texture. So the most common injury associated with a blast wave is a pulmonary contusion. On microscopic examinations the changes closely resemble contusions in non-penetrating blunt chest trauma.

Lung injury by a blast wave occurs when an overpressure of about 40 PSI is reached. Blunt chest trauma leads to a comparable lung contusion, when traumata produce instantaneous changes in velocity when frontal crashes into a fixed object occur as well as near-side lateral impact during a vehicular crash .

Pulmonary contusion from blunt non-penetrating chest trauma is often accentuated to one side and in areas where the thoracic cage is relatively elastic and compressible. Radiographic changes are localized accordingly. Explosion-related trauma, on the other hand, often produces a characteristic "butterfly" pattern in chest CT due to the broad front of the blast wave and reflections of the blast wave if explosions occur in an enclosed space.

We have added in the introduction this interesting point for the reader, and we thank the reviewer for this input. We discussed this subject in less detail, so that we did not too much extend the text.

**Point 6:** Discussion on OP characteristics

In their response, the authors acknowledge they cannot answer the question whether short duration blast OP related injuries are or are not different from longer (and more representative) duration BOP related injuries. This has to be discussed in the manuscript as a limitation.

Thank you very much for noting this issue. We formulated this fact in the limitation section as follows: Furthermore, it is unclear whether shorter and possibly more representative duration blast over pressure related injuries are distinct from longer and more frequent duration blast over pressure related injuries.

**Point 7:** W/D ratio
The information given in the response should benefit to the discussion in the manuscript.For sure, the rise in W/D ration at 15 min is solely due to intra-alveolar hemorrhage, the rise at 3h due to both hemorrhage and edema.

I appreciate your comment in this case. In the related section now we have noted that: Indeed the rise in wet/dry ratio at 15 minutes is solely due to intra-alveolar haemorrhage, while the rise of this ratio at 3 hours could be due to both haemorrhage and oedema.

**Point 8:** OK

**New point**: Can the authors specify at what time control animals were sacrificed?

10 minutes, which is the identical time point as the sacrifice of the first group concerning time points.

**Reference**
